# Supplementary figures and images for: Sepsis-associated neuroinflammation in the spinal cord
Source: PLoS One. 2022 Jun 13;17(6):e0269924. doi: 10.1371/journal.pone.0269924 (PMC9191735; doi:10.1371/journal.pone.0269924)

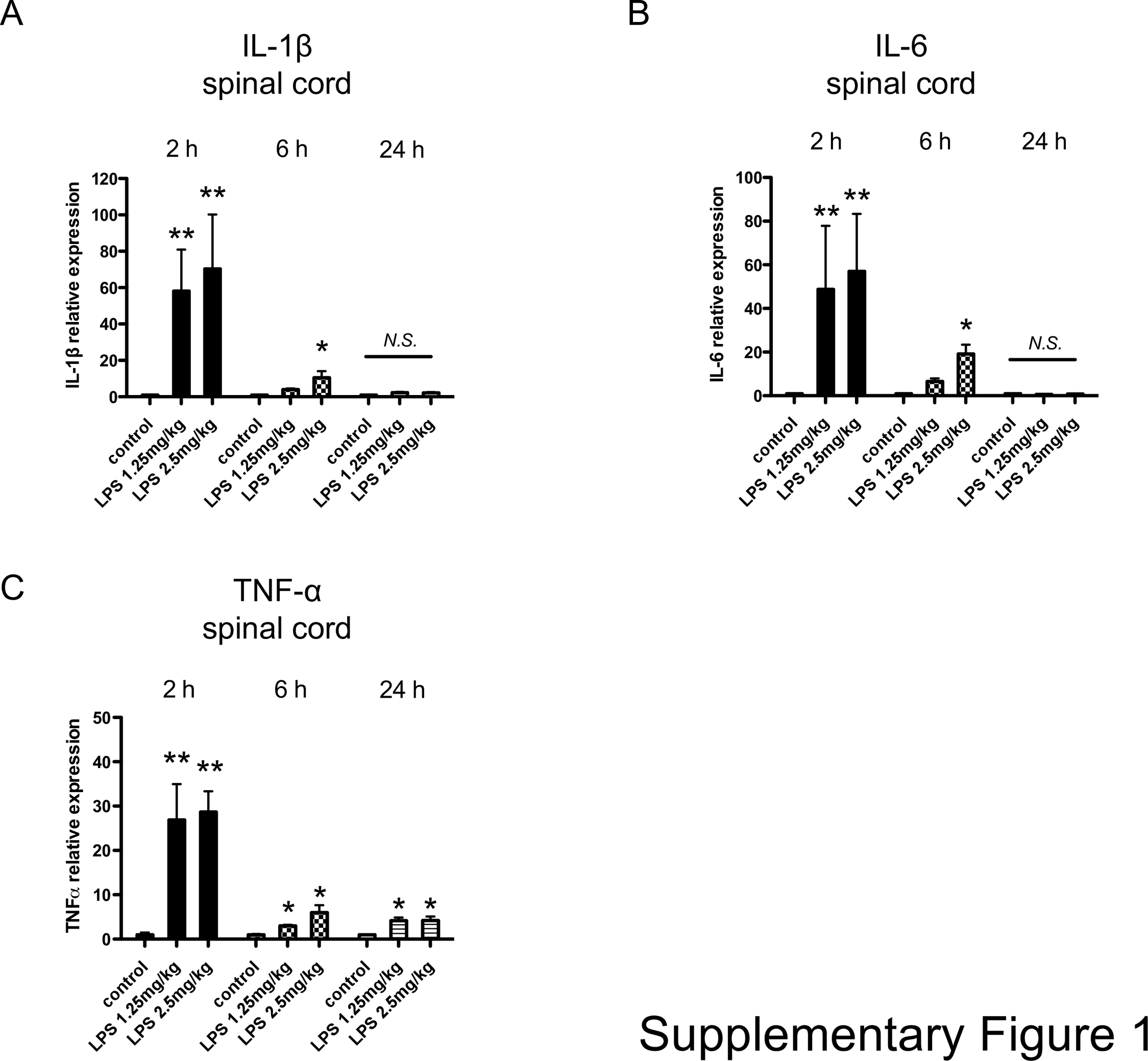

Supplement: S1 Fig — LPS 1.25 or 2.5 mg/kg, or the same amount of normal saline, was intraperitoneally administrated to 10-week-old C57BL6 male mice. mRNA expression levels of pro-inflammatory cytokines were determined in their spinal cords (A-C). mRNA were assayed using real-time quantitative polymerase chain reactions (qRT-PCR; n = 3–5), and the expression levels were normalized to those of 18S rRNA and expressed relative to the mean in control mice. Data are presented as means ± standard deviations (S.D.); *P < 0.05 versus control; N.S., not significant. (TIF) [file pone.0269924.s001.tif]

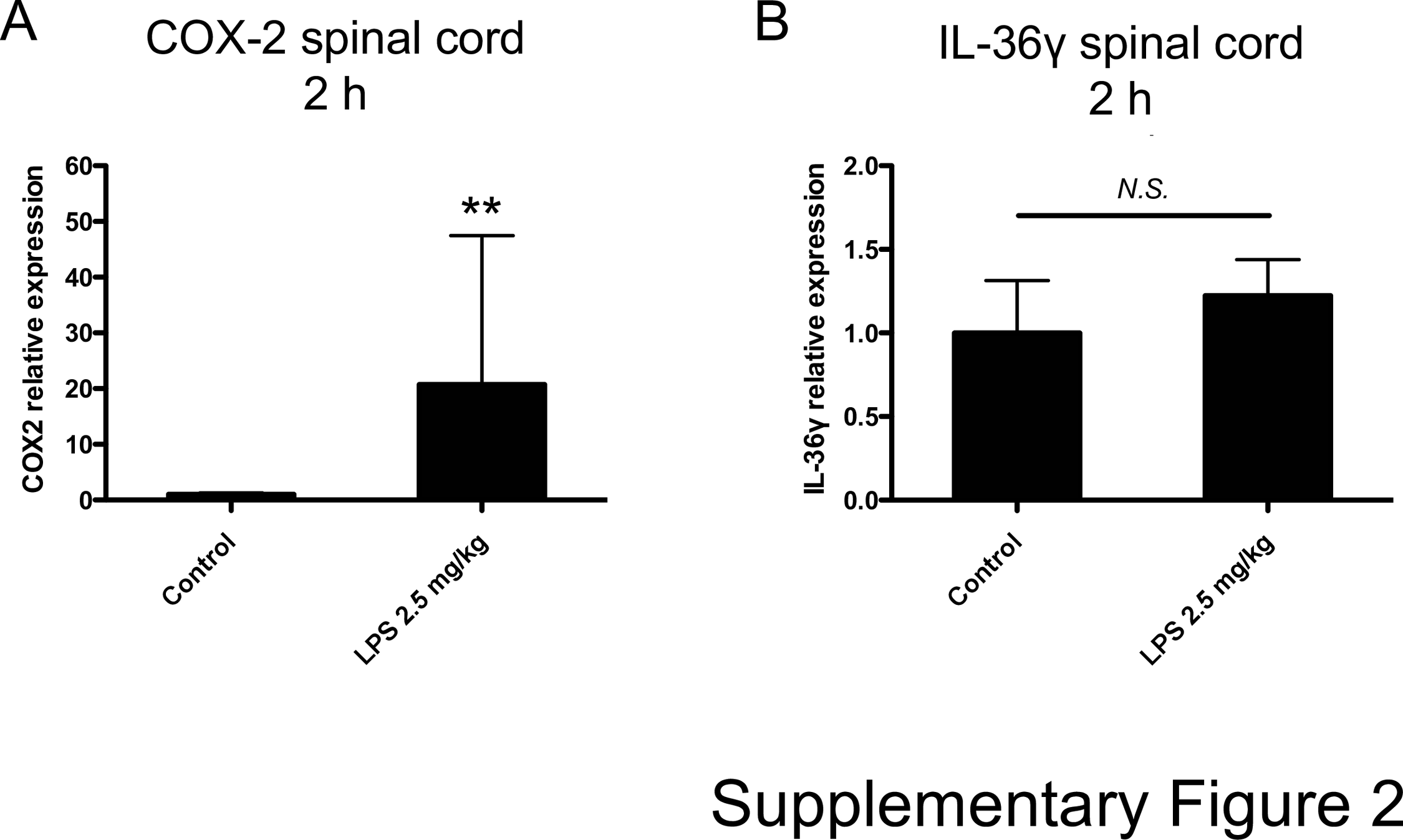

Supplement: S2 Fig — LPS 2.5 mg/kg, or the same amount of normal saline was intraperitoneally administrated to 10-week-old C57BL6 male mice. Pro-inflammatory cytokine COX-2 (A) and IL-36γ (B) mRNA expression were determined in their spinal cords 2 h after LPS administration. MRNA were assayed using real-time quantitative polymerase chain reactions (qRT-PCR; n = 4), and the expression levels were normalized to those of 18S rRNA and expressed relative to the mean in control mice. Data are presented as means ± standard deviations (S.D.); *P < 0.05 versus control; N.S., not significant. (TIF) [file pone.0269924.s002.tif]
